# Incidence of mortality and its predictors among low birth weight neonates in Ethiopia: Systematic review and meta-analysis

**DOI:** 10.1371/journal.pone.0344213

**Published:** 2026-07-29

**Authors:** Wubet Tazeb Wondie, Chalachew Adugna Wubneh, Gebrehiwot Berie Mekonen, Bruck Tesfaye Legesse, Belay Tafa Regassa, Daniel Niguse Mamo, Zenebe Abebe Gebreegziabher, Mariam Alemayehu Abebaw, Gezahagn Demsu Gedefaw, Leweyehu Alemaw Mengstie, Alemu Birara Zemariam

**Affiliations:** 1 Department of Pediatrics and Child Health Nursing, College of Medicine and Health Sciences, Bahir Dar University, Bahir Dar, Ethiopia; 2 Department of Pediatrics and Child Health Nursing, School of Nursing, College of Medicine and Health Science, University of Gondar, Gondar, Ethiopia; 3 Department of Pediatrics and Child Health Nursing, College of Health Science, Debre Tabor University Debre Tabor, Ethiopia; 4 Department of Pediatrics and Neonatal Nursing, School of Nursing and Midwifery, Institute of Health Science, Wollega University, Nekemet, Ethiopia; 5 Department of Medical Laboratory Sciences, College of Health Sciences and Referral Hospital, Ambo University, Ambo, Ethiopia; 6 Department of Health Informatics, School of Public Health, College of Medicine and Health Sciences, Arba Minch University, Arba Minch, Ethiopia; 7 Department of Epidemiology and Biostatistics, School of Public Health, Asrat Woldeyes Health Science campus, Debre Berhan University, Debre Berhan, Ethiopia; 8 Department of Medical Physiology, College of Health Sciences and Referral Hospital, Ambo University, Ambo, Ethiopia; 9 Department of Neonatal Health Nursing, School of Nursing, University of Gondar, Gondar, Ethiopia; 10 Department of Pediatrics and Child Health Nursing, School of Nursing and Midwifery, Asrat Woldeyes Health Sciences campus, Debre Berehan University, Debre Berhan, Ethiopia; 11 Department of Pediatrics and Child Health Nursing, School of Nursing, College of Medicine and Health Science Woldia University, Woldia, Ethiopia; Bangabandhu Sheikh Mujib Medical University (BSMMU), BANGLADESH

## Abstract

**Background:**

Low birth weight neonates are twentyfold at risk of death. Notably, it is a major public health concern in sub-Saharan Africa, including Ethiopia. Despite the presence of several primary studies on this issue in Ethiopia, the study findings are inconsistent. Hence, this review aimed to assess the pooled incidence and proportion of mortality and predictors among low birth weight neonates.

**Method:**

A search of articles from databases (PubMed, CINAHL, Global Index Medicus, and HINAR), and other sources (Google, Google Scholar) were done. All observational studies that assess mortality of low birth weight neonates were included. The Joana Brigs Quality appraisal checklist was used. The pooled incidence rate, proportion, and the effect size of predictors were estimated using a random-effects model. Heterogeneity was assessed using I^2^ test. Subgroup and sensitivity analysis was conducted. Eggers test and funnel plot were used to assess publication bias. Trim and fill analysis was conducted.

**Result:**

Fifteen studies with 7539 study participants were included. The pooled incidence rate of mortality was found to be 36.31 (95 CI: 26.37–46.25) per 1000 neonates’ day observation and the proportion of mortality was 26.67% (95% CI: 21.83–31.51). Preeclampsia (AHR = 1.38; 95% CI: 1.09–1.75), respiratory distress syndrome (AHR: 1.67; 95% CI:1.25–2.23), perinatal asphyxia (AHR: 1.66; 95% CI: 95%:1.35–2.05), sepsis (AHR: 2.04; 95% CI: 1.59–2.63), not breastfeeding (AHR:5.16; 95% CI: 2.61–9.96), not using KMC (AHR: 4.46, 95% CI: 2.19–9.10), extremely low birth weight (AHR: 3.67; 95% CI: 2.55–5.29), very low birth weight (AHR = 1.75; 95% CI: 1.44–2.12), and prematurity (AHR: 1.55; 95% CI: 1.07–2.24) were predictors.

**Conclusion:**

Based on the pooled proportion of mortality, above one-fourth of low birth weight neonates died in Ethiopia, and the incidence rate of mortality was significantly high. Preeclampsia, respiratory distress syndrome, perinatal asphyxia, sepsis, not breastfeeding, not using kangaroo mother care, hypothermia, extremely low and very low birth weight, and prematurity were predictors of mortality. Hence, the concerned stakeholder should focus on LBW neonates presented with these predictors. Maternal and neonatal screening, kangaroo mother care, and breastfeeding need to be strictly practiced to reduce mortality. Furthermore, community-based essential newborn care needs to be strengthened.

**Registration:**

CRD42024524189.

## Background

The mortality of Low Birth Weight (LBW) neonates is a significant global public health concern, particularly in Low, and Middle-Income Countries (LMIC) [1]. Globally, LBW neonates are twentyfold at risk of death relative to normal-weight neonates [2, 3], and in some resource-limited settings, these neonates are more than twenty-five fold at risk of death [4]. Each year, approximately 2.5 million neonates die worldwide, over 80% of whom are low birth weight, with the majority of these deaths occurring in Sub-Saharan Africa (SSA), and Southeast Asia [1,5].

In low-income countries, LBW neonates have low survival probability, and the majority of them develop different sequels [6]. Neonates in SSA, are tenfold at risk of death compared with high-income countries, and the risk is notably high in LBW neonates [7]. As aforementioned above, the magnitude of LBW mortality is high in some regions and countries. For instance, in Southeast Asia, 19%−37%, Eastern Mediterranean 32% [8], in four Asian countries 15% [9], all Asian 23% [10], SSA 21% to 23.9% [11], whole Africa 55% [12], Brazil 12% [4]. Malaysia 34% [13], Italy 15.7% [14] Spain 17% [15], Japan 10% [15], Bangladesh 13.3% [16], Uganda 73.7% [17], and Nigeria 154.5 per 1000 LBW neonates died [18].

As several studies investigated, the predictors of mortality among LBW neonates are being Very LBW [9,17,19-22], 1^st^ & 5^th^ minute Appearance, Pulse, Grimace, Appearance, Respiration (APGAR) score [9,23], Necrotizing Enter colitis (NEC) [9,24], Kangaroo Mother Care (KMC) [25,26], prematurity [19,21,22,27], sepsis [10,13,23,28], hypothermia [13,23, 28], sex, Respiratory Distress Syndrome (RDS), mode of delivery [23], multiple birth [14], and congenital anomaly [10,29]. The mortality of LBW neonates places a substantial burden on families, the national economy, and health care services at the country level [30,31].To avert this worldwide problem, and increase the survival probability of LBW neonates, globally as well as nationally different efforts such as; nutritional provision and counseling for mothers of childbearing age [3], kangaroo mother care (KMC) [32], skin cleansing, and home-based newborn care [33] are implemented.

Ethiopia is one the country with the highest neonatal mortality in the world [34], with neonatal mortality of 33 per 1000 live births [35]. Various studies were conducted on the incidence of mortality among LBW neonates, however, their findings have been highly varied and inconsistent, ranging from 14.5 [36] to 75.63 deaths [37] per 1000 neonates days of observation. Hence, the findings are fragmented and, don’t provide a comprehensive and robust conclusion about the mortality of LBW neonates and its predictors at the national level. Despite the general understanding that LBW neonates are at high risk of death, to the best of our knowledge, there is no comprehensive study that shows the pooled incidence and proportion of mortality among LBW neonates in Ethiopia. Therefore, this systematic review and meta-analysis aimed to assess the incidence and proportion of mortality, and its predictor among LBW neonates in Ethiopia. The findings of this study will help and provide new input for decision-makers and program planners to design appropriate strategies and mobilize resources for the care of more vulnerable neonates at the national level.

## Methods

### Prospero registration and reporting

This systematic review and meta-analysis has been registered in the international prospective registry of Prospero with registration number (CRD42024524189). This review has been reported in accordance with the Preferred Items for Systematic Review and Meta-Analysis (PRISMA 20 statement) guideline [38] (S1 Table).

### Search strategy

A systematic search of literature from databases such as PubMed, CINAHL, Global Index Medicus (GIM), and HINARI with Medical Subject Heading (MeSH) terms and keywords was done. In addition, a manual search of published and unpublished literature from search engines (Google, Google Scholar, Worldwide Science), and Ethiopian University Repositories (University of Gondar, Jimma University, Bahir Dar University, Addis Ababa University, and Debre Berhan University) was done. Moreover, we screened the reference list of included studies, which were deemed important for our study. During the search, we included articles from inception until August 15, 2024. Our search strategy focuses on studies that report the incidence rate, proportion, and predictors of mortality among LBW neonates in Ethiopia. The following key terms, such as: (Incidence OR “epidemiology” OR Occurrence OR Outcome OR Magnitude OR Prevalence OR Burden OR Proportion) AND (Mortality OR Death OR Fatality rate OR Survival OR “Survival rate” OR “Time to death”) AND (Predictors OR “Associated factors” OR Determinant OR “Risk factors”) AND (“Low birth weight neonates” OR “Low birth weight infants” OR “Low birth weight newborn” OR “Very low birth weight neonates” OR “Extremely low birth weight neonates” OR “Small birth weight neonates “OR “Small for gestation age” OR “Small birth weight babies” OR “Small birth weight infants” OR “Small birth weight newborns” OR “Underweight neonates” OR “Below average birth weight neonates”) AND (“Ethiopia”) were used for searching of literature (S2 Table). A Comprehensive search of studies conducted in databases and web search engines between June 3, 2024, to June 15, 2024.

### Eligibility criteria

#### Inclusion criteria.

In this review, all observational studies (cross-sectional, case-control, and cohort studies) that report the mortality of LBW neonates in Ethiopia, and written in the English language were included. Both published and unpublished studies (found in the Ethiopia University repository) studies regarding the mortality of LBW were included. The incorporated studies were within the PECO framework (P = Low birth weight neonates, E = Different predictors of mortality in LBW neonates, C = LBW neonates without those predictors, O = Mortality (Incidence rat, and proportion of mortality).

#### Exclusion criteria.

Studies that don’t contain the necessary information, such as the outcome variable, sample size, and different study populations, and studies that report the mortality of infants beyond the neonatal period were excluded. In addition to this, Case reports, case series, letters to editors, trials, and meeting reports were excluded.

### Outcomes of measurements

This study has three main outcomes. The first two outcomes are the incidence rate and proportion of mortality among LBW neonates. The third outcome is predictors of mortality among LBW neonates in Ethiopia. The pooled effect size of predictors was computed and described in terms of Hazard Ratio (HR) along with 95% Confidence Interval (CI). The incidence rate of mortality was estimated by dividing the total number of new cases of death among LBW neonates by the total number of LBW neonates’ days of follow-up (person-time/person-days of follow-up). In the primary studies, low birth weight was defined based on the WHO definition, as a birth weight less than 2,500 grams regardless of gestational age [2].

### Data extraction

After searching in each database, the articles were imported to EndNote X9, and duplicated studies were removed. Based on the study question & pre-specified inclusion criteria, two reviewers (WTW& BTR) independently screened the title and abstract. After screening the titles and abstracts, the two reviewers accessed the full text and extracted the necessary information from eligible studies. Any disagreement between reviewers was resolved through discussion and consensus. The following necessary information, such as: first author name, year of publication, study region & design, population, sample size, time at risk in days (follow-up period), proportion of mortality, incidence of mortality, and the hazard ratio of predictors were extracted. These information were extracted using an Excel spreadsheet. In case where data were incomplete, two email contacts of the corresponding author were made, and if no data and information were provided, calculations were performed based on the available information.

### Risk of bias and quality assessment

Three reviewers (WTW, ABZ, CAW) independently assessed the quality of the eligible studies using the Joanna Briggs Institute (JBI) critical appraisal checklist [39]. The checklist consists of 11 criteria for a cohort and 8 criteria for a cross-sectional study. These indicators were turned into 100%, and the quality score was graded as high if >80%, medium between 60–80%, and low, < 60%. Any disagreement between reviewers was resolved by discussion. If the disagreement persisted, the fourth author, BTL, was consulted, and the issue was resolved (**S3 File**).

### Assessment of certainty of evidence

The certainty of evidence for pooled estimates (mortality proportion, incidence rate, and significant predictors) was assessed using GRADE guidelines adapted for prognostic meta-analyses of observational studies (Supplementary Table S4). Observational studies start at LOW certainty due to a non-randomized design. Potential downgrades were applied for: risk of bias (JBI checklist), inconsistency (I² > 50% serious), indirectness, imprecision (wide 95% CIs), and publication bias (Egger’s p < 0.05). No upgrades were applied. Certainty ranged from Low to Moderate (**S4 File**).

### Data synthesis and analysis

The Excel data was imported into STATA version 14 for analysis. The pooled incidence rate, proportion of mortality, and effect size of predictors were estimated using a random effect model by assuming the true effect size varies across studies [40]. Heterogeneity between studies was assessed using a forest plot and I^2^ statistics. The I^2^ test statistics of 0, 25–50%, 50–75% & > 75% were interpreted as no, low, moderate, and high heterogeneity, respectively [41]. To identify the potential source of variation, a subgroup analysis was done. To examine the impact of every single study on the overall pooled estimate of mortality, a sensitivity analysis was carried out. The hazard ratio with its corresponding CI was used to estimate the association of significant predictors with mortality. Publication bias was assessed using the funnel plot and Egger’s regression test. Accordingly, statistical non-significance of publication bias was declared at a P-value > 0.05 [42]. To manage publication bias, trim and fill analysis was applied [43]. The result of this meta-analysis was presented using forest plots and tables.

## Results

### Review process and findings

**Fig 1:**
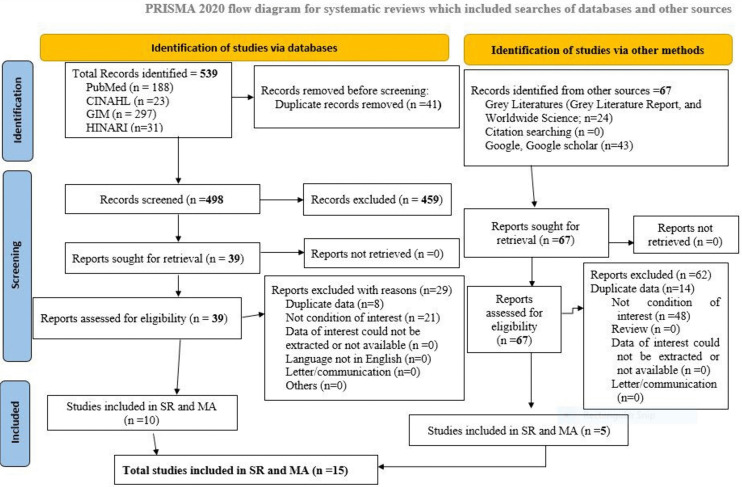
PRISMA 2020 flow diagram for study selection of incidence and proportion of mortality and its predictors among LBW neonates in Ethiopia. SR and MA: Systematic Review and Meta-Analysis.

A total of 606 studies were identified in our search. Of these, 539 were from databases such as (PubMed = 188, CINAHL = 23, Global Index Medicus (GIM)=297, and HINAR = 31). Then, after, 41 of them were found to be duplicated and removed. Four hundred ninety-eight studies remained in the endnote and screened by title and abstract for inclusion, among them, 459 irrelevant studies were excluded. After removing the irrelevant studies, 39 studies were assessed by full text, among them, 29 studies were excluded based on eligibility criteria, and 10 studies were included in the meta-analysis. In addition to these, from other sources (Google, Google Scholar, worldwide science, and unpublished sources), 67 full-text articles were found. Of these, 62 articles that are unrelated to the outcome of interest were excluded, and the remaining 5 articles were included. Therefore, a total of 15 eligible studies were included in this systematic review and meta-analysis (**Fig 1**).

### Characteristics of included studies

After reviewing all the identified studies, 15 (12 published and 3 unpublished) studies conducted in Ethiopia were included. This review covers three regions and one city administration. Accordingly, 6 of them were from the Amhara region, one was conducted in the Oromia region [44]; four were from Addis Ababa [45-48], one was conducted in the Oromia region, and Addis Ababa [49], and three from the Southern Nations Nationalities Peoples Region (SNNPR) [36,50,51]. No studies were reported from the remaining regions of Ethiopia. Regarding study design, four studies were cross-sectional, 7 were retrospective cohort studies, and the rest 4 were prospective cohort studies. The present study included a total of 7539 study participants, and the sample size of the included studies ranged from 161 [46] to 885 [51]. Concerning the magnitude of mortality, the highest mortality was reported in the Amhara region 37.8% [52], and the lowest mortality was reported in SNNPR 8.3% [36]. Similarly, the highest incidence rate of mortality was reported in the Amhara region 75.63 [37], and the lowest incidence rate was reported in SNNPR 14.5 per 1000 neonates day observation [36] (**Table 1**).

**Table 1:** Characteristics of included studies on incidence and predictors of mortality among low birth weight neonates in Ethiopia.

ID	Authors Name	Year of publication	Region	Study design	Sample size	Total follow-up time in days	Number of death	Proportion of death	IR per 1000
1	Wondie et. al,	2023	Amhara	Retrospective follow-up	761	3266	247	32.46	75.63
2	Woelile, et al	2021	Amhara	Retrospective cohort	718	5715	202	28.1	35.3
3	Kebede, et, al	2022	Amhara	Retrospective follow-up	358	3328	126	35.2	37.9
4	Negussie, et, al	2024	SNNP	Prospective follow-up	768	5599	217	28.26	38.8
5	Tessema Z, et, al	2022	Oromia	Retrospective cohort	300	2437.63	85	28.3	34.9
6	Mengstie, et al.	2025	Amhara	Retrospective follow-up	416	2498	107	25.72	42.8
7	Eshete, et, al	2019	SNNP	Cross-sectional	885	**N/R**	97	11	**N/R**
8	Debere, et, al	2022	Oromia &Addis Ababa	Prospective follow-up	808	14967	242	29.95	16.2
9	Dessu, et.al	2020	SNNP	Prospective follow-up	216	1240	18	8.3	14.5
10	Birhanu, et, al	2023	Addis Ababa	Retrospective	329	2608	105	31.9	40.1
11	Genie, et al	2022	Amhara	Retrospective follow-up	291	**NR**	110	37.8	**NR**
12	Abraham	2021	Addis Ababa	Cross-sectional	161	**NR**	37	23	**NR**
13	Gedamu W, et al.	2019	Amhara	Cross-sectional	635	**NR**	161	25.4	**NR**
14	Worku	1999	Addis Ababa	Cross-sectional	604	**NR**	198	32.8	**NR**
15	Mislu, et, al	2024	Addis Ababa	Prospective follow-up	289	**2242**	66	22.84	**29.44**

NR= Not Reported, SNNPR=South Nation Nationality People Region.

### Quality of the included studies

To examine the quality of the included primary studies, the Joanna Briggs Quality (JBI) appraisal checklist for cross-sectional and cohort studies was used. Accordingly, the quality of all included studies in all study designs was from 81.81–100% (S3 File). Additionally, the certainty of evidence was assessed using GRADE for the synthesized evidence detailed in the supplementary file (S4 File)

### Magnitude of mortality among low birth weight neonates

**Fig 2:**
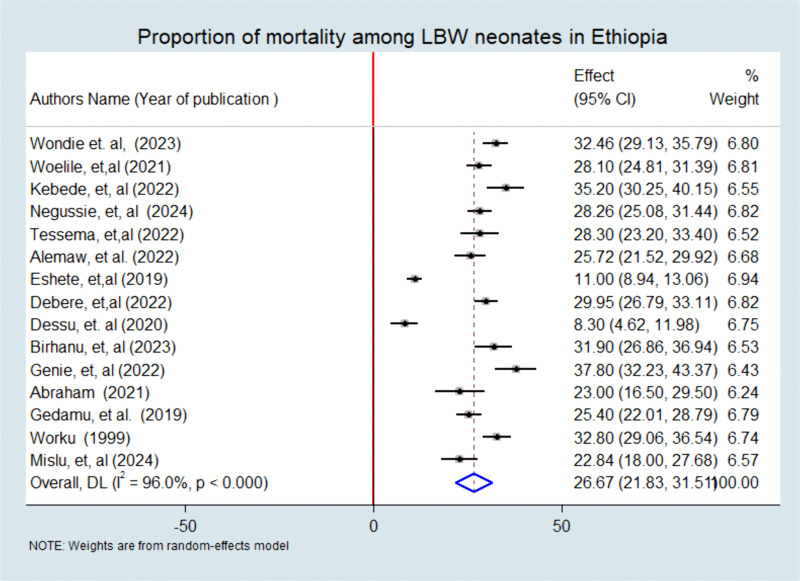
Forest plot of proportion of mortality among low birth weight neonates in Ethiopia.

In this study, to determine the proportion of mortality, fifteen [36,37,44-56] eligible studies were included. In the included studies, the highest proportion of mortality was reported in the Amhara region 37.8% [52], and the lowest proportion of mortality was reported in SNNPR 8.3% [36]. Accordingly, based on the random effect model, the overall pooled proportion of mortality was found to be 26.67% (95% CI: 21.83–31.51). In this study, significant heterogeneity between studies was observed (I^2^ = 96.0%, P < 0.00) (**Fig 2**).

### Handling heterogeneity

In the random effect model, there was significant heterogeneity in the pooled estimate of mortality as verified by I^2^ = 96.0% (P-value = 0.000). To identify the source of potential publication bias, a subgroup analysis and sensitivity analysis were done.

### Sub-group analysis of low birth weight neonates’ mortality

**Fig 3:**
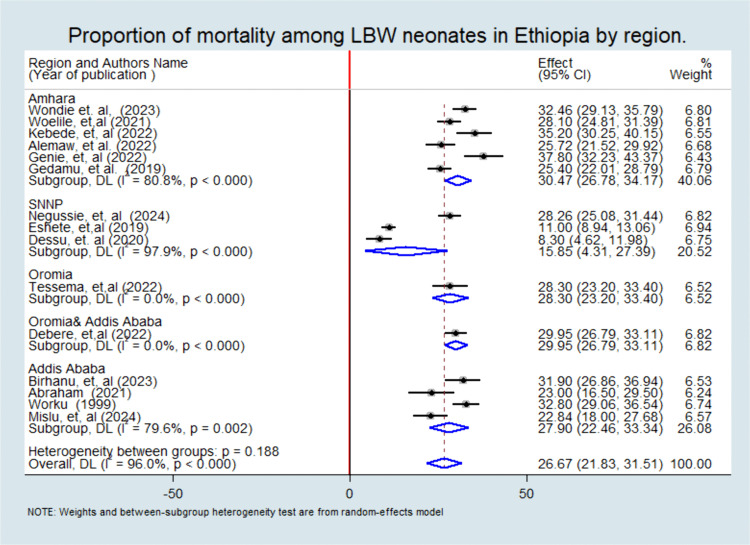
Forest plot of subgroup analysis by region of proportion of mortality among LBW neonates in Ethiopia. SNNP = South Nation and Nationality People Region.

To assess the potential source of heterogeneity, a subgroup analysis based on region was carried out. In the subgroup analysis, the highest proportion of mortality was found in the Amhara region 30.47% (95% CI: 26.78–34.17), and the lowest proportion of mortality was found in SNNP 15.85 (95% CI: 4.31–27.39). As the forest plot showed that there was significant heterogeneity between studies (P-value = 0.02) and in the overall pooled estimate (I^2^ = 96.0%, p-value = 0.00). However, there was no significant heterogeneity between groups (regions) (P-value = 0.188) (**Fig 3**).

### Sensitivity analysis for the proportion of mortality

**Fig 4:**
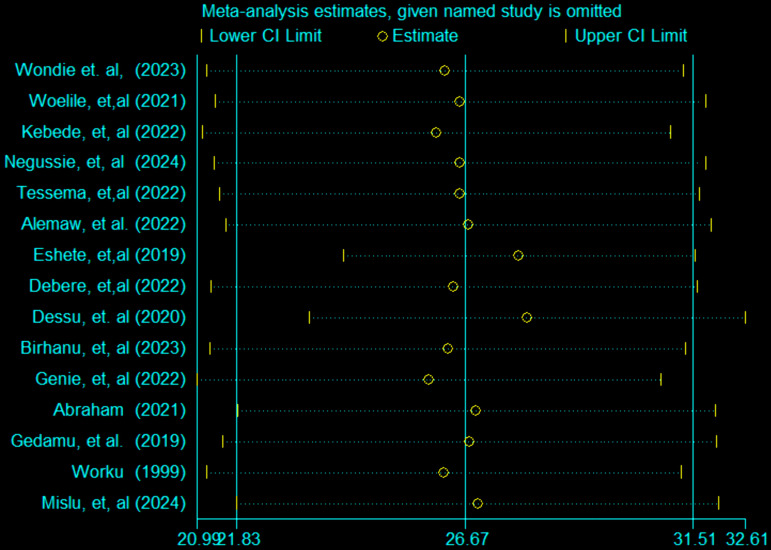
Forest plot showing sensitivity analysis of mortality among low birth weight neonates when studies omitted step by step.

To examine the presence of an influential study on the overall estimate of the proportion of mortality, a sensitivity analysis was done. Accordingly, there was no single study that affected the pooled magnitude of mortality because all the single estimates of the leave-one-out are within the confidence interval of the overall pooled estimate (**Fig 4**).

### Publication bias

**Fig 5:**
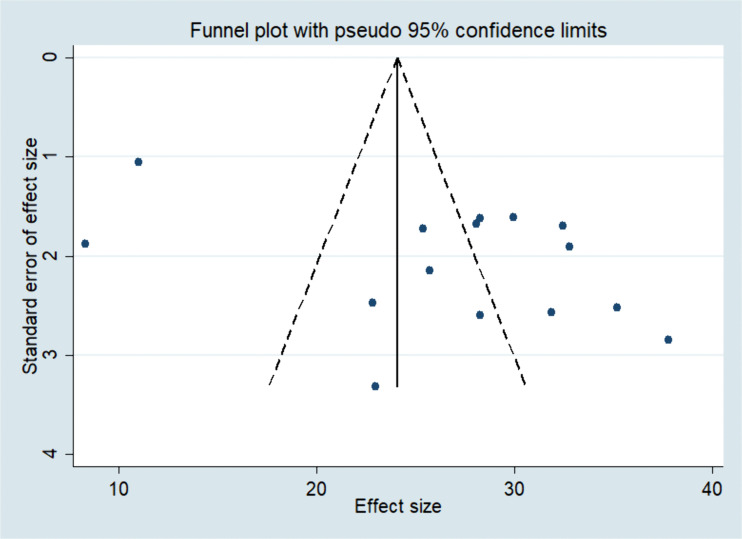
Funnel plot showing publication bias among studies that report the magnitude of mortality among low birth weight neonates.

Publication bias was checked using a funnel plot and Egger’s test. As the funnel plot showed, there was no significant publication bias as confirmed by the symmetrical distribution of the funnel plot (**Fig 5**).

Besides the funnel plot, the Eggers test revealed that there was significant publication bias (P-value = 0.026). To handle this publication bias, a trim and fill analysis was conducted, accordingly, 5 studies were filled, and a total of 20 studies were included in the trim and fill analysis. Based on the random effect model, the pooled magnitude of mortality among low birth weight neonates in the trim and filled analysis was 22.056 (95% CI: 17.39–26.72) (**Table 2**).

**Table 2:** Trim and fill analysis of the pooled estimate of mortality in the filled meta-analysis.

Method	Pooled estimate	95% CI		Asymptomatic		No of studies
		Lower	Upper	Z-value	P-value	20
Fixed	21.043	20.197	21.89	48.761	0.00	
Random	22.056	17.39	26.72	9.268	0.00	

### Incidence of mortality among low birth weight neonates in Ethiopia

**Fig 6:**
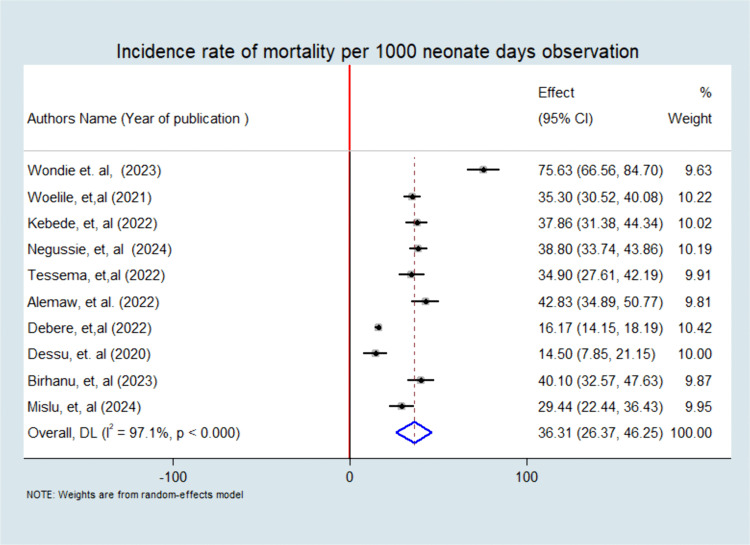
Forest plot showing the incidence rate of mortality per 1000 neonates day observation among low birth weight neonates in Ethiopia.

From the total 15 studies, 10 studies [36,37,44,47-50,53-55] reported the incidence rate of mortality among LBW neonates, and 10 of them were used to report the pooled incidence rate of mortality. Based on the random effect model, the pooled incidence of mortality was found to be 36.31 (95 CI: 26.37–46.25) per 1000 neonates’ day observation with statistically significant heterogeneity (I^2^ = 97.1%, P-value = 0.00) (**Fig 6**).

### Handling heterogeneity

The random effect model showed that there was significant heterogeneity in the pooled incidence rate of mortality. Hence, a subgroup and sensitivity analysis were performed as shown below.

### Sub-group analysis of the incidence rate of mortality among low birth weight neonates

**Fig 7:**
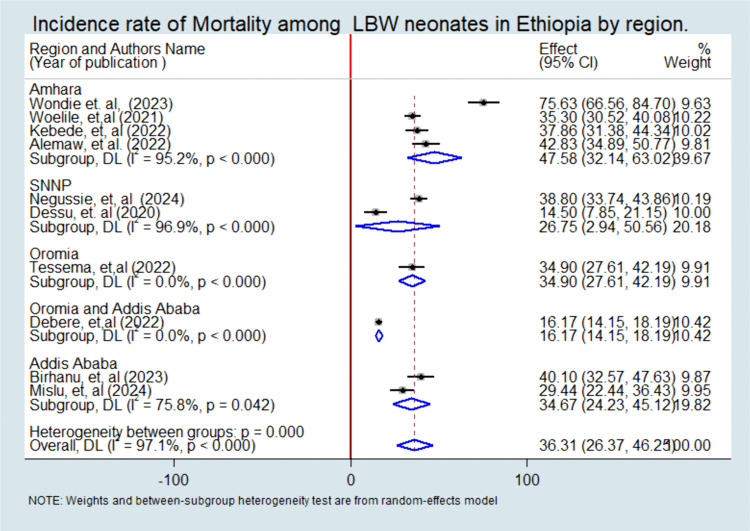
Forest plot showing sub-group analysis of the incidence rate of mortality per 1000 neonates day of observation among low birth weight neonates in Ethiopia.

By considering where the studies were conducted, a subgroup analysis was conducted by region. Accordingly, the random effect model showed that the highest incidence rate of mortality per 1000 neonates per day observation was reported in the Amhara region, 47.58 (95% CI:32.14–63.02), and the lowest incidence rate of mortality was 16.17 (95% CI: 14.15–18.19) per 1000 neonates’ day observation in a study conducted in Oromia and Addis Ababa. In this subgroup analysis, there was significant heterogeneity between studies (P-value < 0.04), and in the overall pooled estimate (p-value = 0.00). However, there was also significant heterogeneity between groups (regions) (P-value = 0.00) (**Fig 7**).

### Sensitivity analysis of incidence rate of mortality

**Fig 8:**
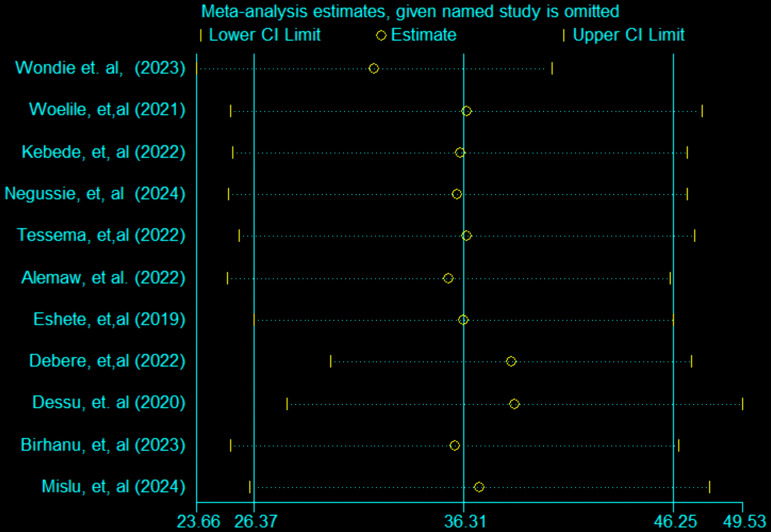
Sensitivity analysis showing incidence rate of mortality among LBW neonates using a random effect model.

Likewise, to the proportion of mortality, a sensitivity analysis to identify the influential studies on the pooled incidence rate of mortality was carried out. However, in the sensitivity analysis, there was no influential study, because all of the single estimates of the leave-one-out analysis were within the confidence interval of the pooled incidence rate mortality, and it was stable (Fig 8).

### Publication bias

**Fig 9:**
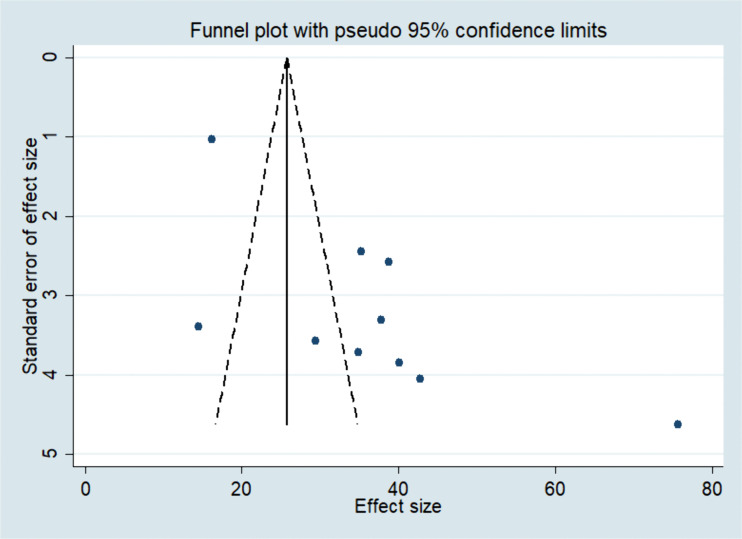
Funnel plot shows publication bias among studies that report the Incidence rate of mortality among Low birth weight neonates in Ethiopia.

The funnel plot for graphical diagnostics of small study effect and Eggers test were used to check publication bias. Accordingly, in the funnel plot for graphical diagnostics of small study effect, there was publication bias as revealed by the asymmetrical distribution of the funnel plot. In addition, the Eggers test revealed that there was significant publication bias (P-value = 0.0155). To handle this publication bias, a trim and fill analysis was applied. (Fig 9).

In addition, the Eggers test revealed that there was significant publication bias (P-value = 0.005). To handle this publication bias, a trim and fill analysis was applied. Accordingly, in the trim and fill analysis, 5 studies were filled and a total of 20 studies were included in the filled meta-analysis. The random effect model revealed that the pooled incidence rate of mortality among LBW neonates in the filled meta-analysis was 22.056 (95% CI; 17.39–26.72) (Table 3).

**Table 3:** Trim and fill analysis of the pooled incidence rate of mortality in the filled meta-analysis.

Method	Pooled estimate	95% CI		Asymptomatic		No of studies
		Lower	Upper	Z-value	P-value	15
Fixed	20.227	18.900	21.555	29.866	0.00	
Random	22.175	12.332	32.019	4.415	0.00	

### Predictors of incidence of mortality among LBW neonates

In this study, the pooled estimated effect size of preeclampsia, PNA, RDS, NEC, prematurity, ELBW, VLBW, hypothermia, Intrauterine Growth Restriction (IUGR), mode of delivery, antenatal corticosteroid, antepartum hemorrhage, 1^st^&5^th^ minute APGAR score, Place of delivery, ANC, Sepsis, Placenta abruptio, maternal illness, Congenital anomaly, Hypoglycemia, Multiple pregnancy, breastfeeding, and KMC on mortality were assessed. However, in the pooled estimate only 10 variables were significant predictors of mortality as stated below.

### Preeclampsia

**Fig 10:**
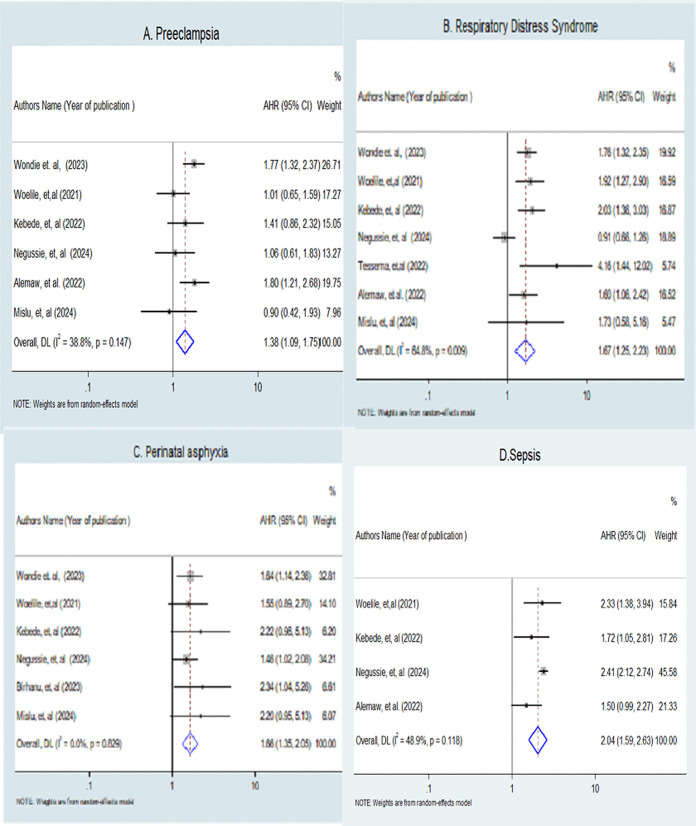
Forest plot showing the effect size of predictors of mortality among LBW neonates in Ethiopia. A): Preeclampsia, B): Respiratory Distress Syndrome, C): Perinatal Asphyxia, and D): Sepsis.

Six studies [37,48,50,53–55] assessed the association of preeclampsia with mortality among LBW neonates, of these, three studies [48,50,54,55] showed that no significant association between preeclampsia and mortality exists. However, the pooled effect size showed that neonates from preeclampsia mothers were 1.38 times at hazard of death compared with their counterparts (AHR = 1.38, 95% CI: 1.09–1.75). From the random effect model, no significant heterogeneity was observed between studies (I^2^ = 38.8%, P-value = 0.147) (Fig 10A).

### Respiratory Distress Syndrome (RDS)

Seven studies [37,44,48,50,53–55] reported the association between RDS and mortality among LBW neonates, among them, two studies [48,50] revealed no association between RDS and mortality among LBW neonates. In the pooled estimate, LBW neonates presented with RDS were 1.67 times at hazard of death compared with their counterparts (AHR: 1.67, 95% CI: 1.25–2.23). The heterogeneity test showed that there was significant variation between studies (I^2^ = 64.8, P-value = 0.009) (Fig 10B)

### Perinatal Asphyxia (PNA)

Six studies [37,47,48,50,54,55] revealed the association between PNA and mortality among LBW neonates. The current study showed that neonates who had PNA had a 1.66 times hazard of death compared with neonates without PNA (AHR: 1.66, 95%:1.35–2.05). Among studies, there was no significant heterogeneity (I^2^ = 0.00, P-value = 0.829 (Fig 10C).

### Sepsis

Four studies were included to assess the association between sepsis and mortality among LBW neonates. One study [53] showed no significant association between sepsis and mortality, while the rest three [50,54,55] showed a significant association. However, in the pooled estimate, LBW neonates presented with sepsis had a twofold hazard of death compared with neonates with no sepsis (pAHR: 2.04; 95% CI: 1.59–2.63). In the random effect model, there was no statistically significant heterogeneity between studies (I^2^ = 48.9, P-value = 0.118) (Fig 10D).

### Not breastfeeding

**Fig 11:**
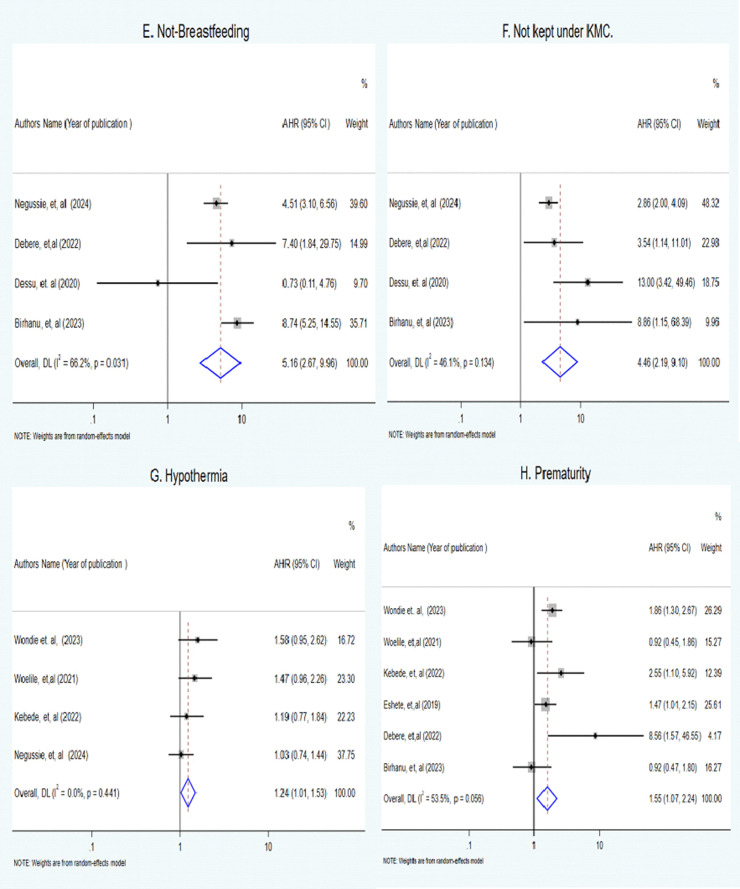
Forest plot of the effect size of predictors of mortality among LBW neonates in Ethiopia. E): Non-Breastfeeding, F): Not kept under KMC, G): Hypothermia, and H): Prematurity.

Four studies [36,47,49,50] revealed the association between not being breastfed and mortality among LBW neonates. The present meta-analysis showed that neonates who are not breastfed had 5.16 times at hazard of death compared with neonates who are breastfeeding (pAHR:5.16, 95% CI: 2.61–9.96). There was moderate heterogeneity across studies (I^2^ = 66.2%, P-value = 0.031) (Fig 11E).

### Not using Kangaroo Mother Care (KMC)

Four studies [36,47,49,50] reported the association between not being kept under KMC and mortality. This meta-analysis showed neonates who are not kept under KMC were 4.46 times at hazard of death compared with their counterparts (AHR: 4.46; 95% CI: 2.19–9.10). The heterogeneity test showed that there was insignificant heterogeneity across studies (I^2^ = 46.1%, P-value = 0.134) (Fig 11F).

### Hypothermia

A total of five studies [37,50,54,55] were included to assess the association between hypothermia and mortality. Accordingly, the present study showed that neonates presented with hypothermia were 1.24 times at hazard of death compared with their counterparts (AHR = 1.24; 95% CI:1.01–1.53). The heterogeneity test revealed that there was no variation across studies (I^2^ = 0.00, P-value = 0.441) (Fig 11G).

### Prematurity

Six studies [37,47,49,51,54,55] were included to assess the association between prematurity and mortality. Among them, two studies [47,55] showed no significant association. However, in the pooled estimate, premature neonates had a 1.55-fold higher risk of death compared with term LBW neonates (AHR: 1.55; 95% CI:1.07–2.24). The heterogeneity test revealed that there was no statistical difference across studies (I^2^ = 53.5, P = 0.056) (**Fig 11H**).

### Extremely low birth weight

**Fig 12:**
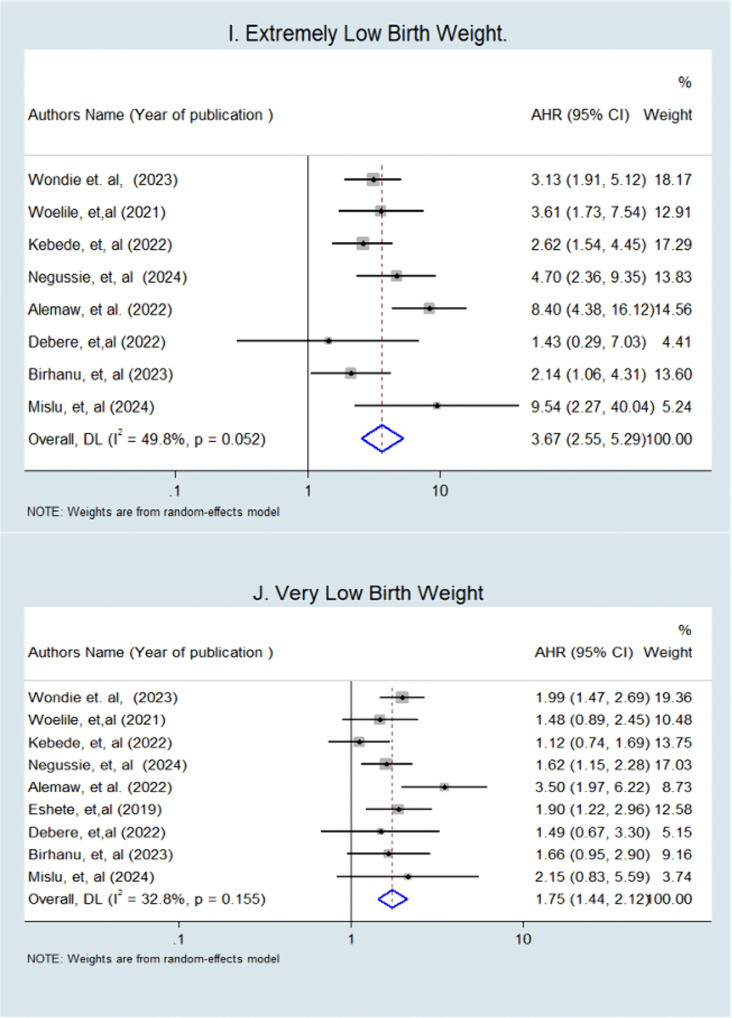
Forest plot showing the effect size of predictors of mortality among LBW neonates in Ethiopia. I): Extremely Low Birth Weight, J): Very Low Birth Weight.

To examine the association of extremely low birth weight neonates (ELBW) with mortality, eight studies [37,47-50,53–55] were included, among them one study [49] showed an insignificant association. The present study showed that being ELBW increases the hazard of death 3.67 times compared with neonates of birthweight 1500–2499 gram (AHR: 3.67 95% CI: 2.55–5.29). In the random effect model, there was no significant heterogeneity across studies (I^2^ = 49.8, P-value = 0.052) (**Fig 12I**).

### Very low birth weight neonate

A total of nine [37,47-51,53-55] studies reported the association between being very low birth weight (VLBW) and mortality. Of them, five studies [47-49,54,55] showed insignificant association. In the current study, the pooled estimate showed that VLBW neonates are nearly twofold at risk of death compared with neonates with birth weight 1500–2499 (AHR = 1.75; 95% CI: 1.44–2.12). In the random effect model, there was no significant heterogeneity (I^2^ = 32.8, P-value = 0.155) (**Fig 12J**)

In general, in this study, Preeclampsia, RDS, PNA, Sepsis, not breastfeeding, not keeping under KMC, hypothermia, ELBW, VLBW, and Prematurity were predictors for the incidence of mortality (Table 4).

**Table 4:** Summary of pooled effect size of predictors of mortality among LBW neonates in Ethiopia.

Variables	No of studies	PHR, 95% CI	Heterogeneity test	
			I2	P-value
Preeclampsia	6	1.38 (1.09-1.75)	38.8	0.147
RDS	7	1.67 (1.25-2.23)	64.8	0.009
PNA	6	1.66 (1.35-2.05)	0.00	0.829
Sepsis	4	2.04 (1.59-2.63)	48.9	0.118
Not breastfeeding	4	5.16 (2.61-9.96)	66.2	0.031
Not keeping under KMC	4	4.46 (2.19- 9.10)	46.1	0.134
Hypothermia	4	1.24 (1.01-1.53)	0.00	0.441
ELBW	8	3.67 (2.55-5.29)	49.8	0.052
VLBW	9	1.75 (1.44- 2.12)	32.8	0.155
Prematurity	6	1.55 (1.07-2.24	53.5	0.056

ELBW; Extremely Low Birth weight, KMC; Kangaroo Mother Care, PNA; Perinatal Asphyxia, VLBW; Very Low Birth Weight, RDS; Respiratory Distress Syndrome, PHR; Pooled Hazard Ratio.

### GRADE summary of findings

The certainty of pooled estimates ranged from LOW to MODERATE (S4 File). Main outcomes (mortality proportion/incidence) were rated LOW primarily due to serious inconsistency (I² > 96%) and publication bias. Predictors showed variable certainty: MODERATE for PNA, hypothermia, ELBW, VLBW (limited inconsistency); LOW for Preeclampsia, RDS, sepsis, not breastfeeding, not keeping under KMC, and prematurity (inconsistency/imprecision) (S4 File).

## Discussion

This systematic review and meta-analysis assessed the incidence rate and proportion of mortality and its predictors among low birth weight neonates in Ethiopia. This study showed that the incidence rate of mortality among LBW neonates in Ethiopia was 36.31 (95 CI: 26.37–46.25) per 1000 neonates’ day observation. This high incidence of mortality might be due to the high number of home delivery [57], lack of community-based essential newborn care practices, maternal undernutrition, low ANC visits, and failure of early detection & management of maternal health issues. Additionally, lack of advanced/specialized neonatal care in resource-limited settings, such as surfactant therapy, Continuous Positive Airway Pressure (CPAP), incubators, and specialized bedding for premature/LBW neonates, likely contributes to the high mortality rate [58]. In the sub-group analysis, the highest incidence rate of mortality was found in the Amhara region, at 47.58 per 1,000 neonate-days (95% CI:32.14–63.02. This notable variation may be due to some distal factors, such as differences in ANC service utilization and poor healthcare-seeking behavior. For instance, ANC visits & service utilization among pregnant mothers in the Amhara region is low [59]. Furthermore, since the majority of the included studies were conducted in Amhara region, the mortality rate of LBW neonates in other regions may not have been adequately assessed and accurately estimated.

This study also assessed the proportion of mortality. Accordingly, this study found that the pooled proportion of mortality among LBW neonates in Ethiopia was 26.67% (95% CI: 21.83–31.51). This finding is in line with a study conducted in SSA (21% −23.9) [11], and the Eastern Mediterranean region (32%) [8]. The possible justification for this might be due to the presence of similar co-morbidities in the source population. In addition to this, the treatment setup of SSA is almost similar to that of Ethiopia. Moreover, it might be due to similarities in the included study design. On the other hand, this finding is lower than studies conducted in Uganda (73.7%) [17] and Malaysia (37.4%) [13]. This might be due to differences in inclusion criteria. Those studies included only VLBW neonates; these neonates are more at risk of death than the other LBW groups. The other reason is the study setting, those studies are based on a national demographic health survey, in this case, neonates in the community who didn’t get appropriate services and at high risk of death were included. However, in the present meta-analysis, the majority of included studies were institution-based.

In addition to the aforementioned variation, the finding of the present study was higher than a study conducted in Italy (15.7%) [14], Spain (17%) [15], Japan (10%) [15], in four Asian countries (15%) [9], Bangladesh (13.3%) [16], and Nigeria (15.5%) [18]. This might be due to differences in inclusion/exclusion criteria. Those studies exclude neonates with lethal malformation, however, in this review, the incorporated studies include neonates with any type of congenital malformation, which may increase the mortality rate. Besides, it might be due to differences in treatment setup and the availability of advanced treatments. For instance, in those studies, very LBW and critically ill neonates were treated at the tertiary level, and they included neonates in Urban setting. But, in Ethiopia all VLBW & critically ill neonates are not treated at the tertiary level, except in some occasions, and advanced treatment like surfactant therapy for preterm neonates is not available. Hence, this factors may affect the survival rate of neonates in Ethiopia. Furthermore, it might be due to socioeconomic disparities between Ethiopia, and those countries, which may increase LBW and subsequent mortality.

Furthermore, this study also assessed predictors of mortality among LBW neonates in Ethiopia. Accordingly, neonates from preeclampsia mother had a higher hazard of death compared with their counterparts. This finding is supported by a study conducted in North America [60]. This is because preeclampsia impairs placental function, which decreases oxygen & nutrient supply. The second reason might be; that preeclampsia necessitates the delivery of the baby before full term for the health of the mother. In addition, preeclampsia increases the risk of IUGR and preterm-related complications, which result in death [61]. Similarly, in this study, RDS was an independent predictor of LBW neonate mortality, this finding is consistent with a study conducted in Brazil [23]. This is due to the fact that, RDS compromises oxygenation and causes respiratory failure, which increases the risk of hypoxemia in vital organs, resulting in organ dysfunction and death [58]. Besides, LBW neonates are born prematurely with immature organs. In the combination of organ immaturity with RDS, neonates can’t overcome respiratory difficulties, which results in death [62].

This study also revealed that PNA is an independent predictor of mortality among LBW neonates. This might be due to the fact that PNA causes multiple organ damage, and LBW neonates are more vulnerable due to immature organs. The additional stress of PNA in LBW neonates exacerbates organ dysfunction, which results in death [58, 62]. In addition, PNA causes hypoxic-ischemic encephalopathy (HIE), which leads to neurological impairment and increases the risk of mortality. Besides, PNA increases the risk of sepsis by weakening the immune system, hence, the synergetic effect of PNA & sepsis in LBW neonates increases the risk of mortality. In agreement with studies conducted in the Eastern Mediterranean region [10], Brazil [24], China [63], Malaysia [64], and one global review [65], sepsis was a significant predictor of mortality. This might be due to delayed diagnosis and difficulties in treatment. In LBW neonates, diagnosing sepsis is more challenging, and their treatment is delayed, which shortens their survival [58]. Besides, sepsis induces multi-organ dysfunction in LBW neonates due to organ immaturity, which results in death [66].

In the present study, non-breastfeeding neonates are more than fivefold at hazard of death. This finding is supported by a study conducted in Pelotas and Southern Brazil [67], and meta-analysis [68]. The possible justification might be the lack of different antibodies in breast milk that have antimicrobial activity, this induces different comorbidity and ends up with mortality. On the other hand, lack of breastfeeding compromises the nutritional status of the neonates [1,62]. In addition, formula feeding for non-breastfeeding neonates introduces infection and allergy due to unhygienic conditions, which increases the risk of mortality [58,62]. As studies in the low and middle-income countries [33], and Pakistan [26] revealed, LBW neonates not kept under KMC are at hazard of death. The present study also revealed LBW neonates not kept under KMC were more than fourfold at the hazard of death. The possible reason might be that LBW neonates have limited body fat for thermoregulation, but KMC provides thermal regulation. Without KMC, these LBW neonates develop hypothermia, which increases other comorbidities and leads to death [58,69]. On the other hand, KMC promotes breastfeeding, hence not using KMC increases the risk of infection in LBW neonates [62,69]. Besides, not keeping LBW neonates under KMC affects mother-neonate bonding [62].

In agreement with studies conducted in Malaysia [13], Brazil [24], and a meta-analysis [28]. In this study, hypothermia increases, the hazard of death. This might be due to poor perception of hypothermia as a risk factor for death, even among health professionals in low-income countries [70]. In addition, it might be due to inappropriate use of incubators [71].

The current study also revealed that extremely low birth weight (ELBW) & very low birth weight (VLBW) neonates were more than threefold and nearly twofold at the hazard of death, respectively. This finding is consistent with studies conducted in Asia [9], Uganda [17], Zambia [19], and Brazil [20]. This is due to the fact that ELBW & VLBW neonates have immature organ systems, and they are more vulnerable to respiratory distress syndrome, necrotizing enterocolitis, and hypothermia, and the majority of them are premature, which increases the hazard of death. On the other hand, ELBW& VLBW are at risk of neurological complications such as periventricular leukomalacia, and intraventricular hemorrhage, which shortens the survival of neonates [72]. Besides, ELBW& VLBW neonates may expose to different invasive procedures, and may have long hospital stay, these factors increase the risk of severe systemic infection, which leads to death [62,73].

In agreement with studies conducted in East Africa [27], Netherland [74], Zambia [19], and systematic review [22], prematurity is a significant predictor of mortality among LBW neonates in Ethiopia. The possible reason might be that premature neonates have immature/underdeveloped organ systems to maintain normal homeostasis, which increases the risk of respiratory distress, hypothermia, hypoglycemia, and infection these factors shorten the survival probability of neonates [75]. On the other hand, premature LBW neonates have feeding difficulty, which causes inadequate nutrition, this problem affects their immune function, and overall heath, which leads to death [76]. In addition to the aforementioned reasons, premature LBW neonates are more susceptible to necrotizing enterocolitis, which increases the hazard of death [63].

### Strength and limitations

This is the first review that showed the pooled incidence and proportion of mortality and its predictors in Ethiopia. Despite this, this study doesn’t include all regions of the country due to the limited availability of studies, so it may lack national representativeness. In addition, some predictors, like maternal age and parity, were not assessed because the primary studies categorize those variables differently. Hence, we recommend further study.

## Conclusions

Based on the pooled proportion of mortality, more than one-fourth of low birth weight neonates in Ethiopia have died. The incidence rate of mortality among low birth weight neonates was significantly higher, strikingly elevated compared to that of normal-weight neonates. Preeclampsia, RDS, PNA, sepsis, not breastfeeding, not using KMC, hypothermia, ELBW, VLBW, and prematurity increase the hazard of death.

The preventable death of LBW neonates requires urgent attention. To address this issue, it is essential to focus specifically on these high-risk groups of neonates. Hence, the concerned stakeholder needs to give special care and emphasis to those LBW neonates presented with the identified predictors. In particular, early maternal and neonatal screening, along with the promotion and strict implementation of Kangaroo Mother Care (KMC) and breastfeeding, must be strengthened to address contributing factors and reduce mortality rates. In addition, community-based essential newborn care and support for the families of LBW neonates should be provided and strengthened.

## Supporting information

S1 Table The preferred Items of Systematic Review and Meta-analysis checklist for reporting Incidence and proportion of mortality and its predictors among LBW neonates in Ethiopia. (DOCX)

S2 Table Search terms and strategies for mortality and predictors among LBW neonates in Ethiopia. (DOCX)

S3 Table Quality assessment of included studies using JBI Quality appraisal checklist. (DOCX)

S4 Table Certainty of evidence according to GRADE Approach. (DOCX)

## Abbreviations and Acronyms:

ANC: Antenatal Care
APGAR: Appearance, Pulse,
Grimace: Activity, Pulse
ELBW: Extremely Low Birth Weight
IUGR: Intrauterine Growth Restriction
LBW: Low Birth weight
NEC: Necrotizing Enterocolitis
PNA: Perinatal Asphyxia
RDS: Respiratory Distress Syndrome
SNNPR: South Nation Nationality Region
SSA: Sub-Saharan Africa
SR and MA: Systematic Review and Meta-Analysis
VLBW: Very Low Birth Weight
