# Supplementary material for: Incidence of mortality and its predictors among low birth weight neonates in Ethiopia: Systematic review and meta-analysis
Source: PLoS One. 2026 Jul 29;21(7):e0344213. doi: 10.1371/journal.pone.0344213 (PMC13419215; doi:10.1371/journal.pone.0344213)
Supplement: S2 Table — (DOCX) [file pone.0344213.s002.docx]

**S2 Table: Search Terms for PubMed, CINHAL, Global Index Medicus (GID).**

**Option -1.**

|  | Concept 1 | Concept 2 | Concept 3 | Concept 4 | Concept 5 | Concept 6 |
| --- | --- | --- | --- | --- | --- | --- |
| Free text/word | Incidence | Mortality | Predictors | “Low birth weight” | Neonates | Ethiopia |
| Mesh | “epidemiology” | - | - | - | “infant,  newborn” | “ethiopia” |
| Related Terms | Occurrence  Outcome  Magnitude  Prevalence  Burden  Proportion  Predictors  “Associated factors”  Determinant  “Risk factors” | Death  Fatality rate  Survival  “Survival rate”  “Time to death“ | “Associated factors”  Determinant  “Risk factors” | “Very low birth weight”  “extremely low birth weight”  “Small birth weight “  “Small for gestation age”  “Reduced birth weight”  “Light birth weight” “Below average birth weight” | Newborns  Infant  Babies  “Neonatal babies”  “New born babies”  “Newborn infant”  “Neonatal infants” | Addis Ababa”  “Dire Dawa”  “Benishangul-Gumuz”  Afar  Oromia  Somali  Tigray  Gambella  Harari  “Southern nations, nationalities and peoples’ region”  SNNPR  Amhara  “Sidama region” |
|  |  |  |  |  |  |  |

Option -2

|  | Concept 1 | Concept 2 | Concept 3 | Concept 4 |
| --- | --- | --- | --- | --- |
| Free text/word | Incidence | Mortality | “Low birth weight neonates” | Ethiopia |
| Mesh |  |  |  |  |
| Related Terms | Occurrence  Outcome  Magnitude  Prevalence  Burden  Proportion  Predictors  “Associated factors”  Determinant  “Risk factors”  Time to death | Death  “Fatality rate”  Survival  “Survival rate”  “Time to death” | “Low birth weight infants”  “Low birth weight new born”  “Very low birth weight neonates”  “Extremely low birth weight neonates”  “Small birth weight neonates “  - “Small for gestation age”  “Small birth weight babies”  “Small birth weight infants”  “Small birth weight newborns”  - “Underweight neonates”  - “Below average birth weight neonates” | Addis Ababa”  “Dire Dawa”  “Benishangul-Gumuz”  Afar  Oromia  Somali  Tigray  Gambella  Harari  “Southern nations, nationalities and peoples’ region”  SNNPR  Amhara  “Sidama region” |
|  |  |  |  |  |

**Pubmed Total result (n=188)**

**Search: ((((((((((((((((Incidence[Title/Abstract]) OR ("epidemiology"[Subheading])) OR ("incidence"[MeSH Terms])) OR (Occurrence[Title/Abstract])) OR (Outcome[Title/Abstract])) OR (Magnitude[Title/Abstract])) OR (Prevalence[Title/Abstract])) OR (Burden[Title/Abstract])) OR (Proportion[Title/Abstract])) OR (Predictors[Title/Abstract])) OR ("Associated factors"[Title/Abstract])) OR (Determinant[Title/Abstract])) OR ("Risk factors"[Title/Abstract])) AND ((((((Mortality[Title/Abstract]) OR (Death[Title/Abstract])) OR ("Fatality rate"[Title/Abstract])) OR (Survival[Title/Abstract])) OR ("Survival rate"[Title/Abstract])) OR ("Time to death"[Title/Abstract]))) AND (((((((("Low birth weight"[Title/Abstract]) OR ("Very low birth weight"[Title/Abstract])) OR ("extremely low birth weight"[Title/Abstract])) OR ("Small birth weight "[Title/Abstract])) OR ("Small for gestation age"[Title/Abstract])) OR ("Reduced birth weight"[Title/Abstract])) OR ("Light birth weight"[Title/Abstract])) OR ("Below average birth weight"[Title/Abstract]))) AND (((((((((Neonates[Title/Abstract]) OR ("infant, newborn"[MeSH Terms])) OR (Newborns[Title/Abstract])) OR (Infant[Title/Abstract])) OR (Babies[Title/Abstract])) OR ("Neonatal babies"[Title/Abstract])) OR ("New born babies"[Title/Abstract])) OR ("Newborn infant"[Title/Abstract])) OR ("Neonatal infants"[Title/Abstract]))) AND (((((((((((((((Ethiopia[Title/Abstract]) OR ("ethiopia"[MeSH Terms])) OR ("Addis Ababa"[Title/Abstract])) OR ("Dire Dawa"[Title/Abstract])) OR ("Benishangul-Gumuz"[Title/Abstract])) OR (Afar[Title/Abstract])) OR (Oromia[Title/Abstract])) OR (Somali[Title/Abstract])) OR (Tigray[Title/Abstract])) OR (Gambella[Title/Abstract])) OR (Harari[Title/Abstract])) OR ("Southern nations, nationalities and peoples’ region"[Title/Abstract])) OR (SNNPR[Title/Abstract])) OR (Amhara[Title/Abstract])) OR ("Sidama region"[Title/Abstract]))**

**Global Index Medicus (n=297)**

**(tw:(Incidence)) OR (tw:(Occurrence)) OR (tw:( Outcome)) OR (tw:(Magnitude)) OR (tw:(Prevalence )) OR (tw:(Burden)) OR (tw:(Proportion )) OR (tw:( Predictors)) OR (tw:(“Associated factors” )) OR (tw:(Determinant )) OR (tw:(“Risk factors” )) AND (tw:(death )) OR (tw:(Mortality )) OR (tw:(Fatality rate)) OR (tw:( Survival )) OR (tw:( “Survival rate”)) OR (tw:(“Time to death“ )) AND (tw:( “low birth weight neonates” )) OR (tw:(“Very low birth weight neonates” )) OR (tw:(“Extremely low birth weight neonates”))**

**HINARI (n=31)**

**(Incidence OR Magnitude OR Prevalence OR Proportion OR Predictors OR “Associated factors” OR Determinant OR “Risk factors”) AND (Mortality OR Death OR Survival OR “Survival rate” OR “Time to death") AND (“Low birth weight neonates” OR “Low birth weight new born” OR “Very low birth weight neonate" OR "small for gestational age") AND (Ethiopia).**

**CINAHL(n=23)**

**(Incidence OR Occurrence OR Outcome OR Magnitude OR Prevalence OR Burden OR Proportion OR Predictors OR “Associated factors” OR Determinant OR “Risk factors”) AND (Mortality OR Death OR “Fatality rate” OR Survival OR “Survival rate” OR “Time to death”) AND (“Low birth weight neonates” OR “Low birth weight infants” OR “Low birth weight new born” OR “Very low birth weight neonates” OR “Extremely low birth weight neonates” OR “Small birth weight neonates “ OR “Small for gestation age” OR “Small birth weight babies” OR “Small birth weight infants” OR “Small birth weight newborns” OR “Underweight neonates” OR “Below average birth weight neonates”).**
