# Supplementary material for: Incidence of mortality and its predictors among low birth weight neonates in Ethiopia: Systematic review and meta-analysis
Source: PLoS One. 2026 Jul 29;21(7):e0344213. doi: 10.1371/journal.pone.0344213 (PMC13419215; doi:10.1371/journal.pone.0344213)
Supplement: S3 Table — (DOCX) [file pone.0344213.s003.docx]

S3 Table A: Quality assessment for included cohort studies in meta-analysis for Cohort study

| Author, Year | Q1 | | | | Q2 | | | | Q3 | | | | Q4 | | | | Q5 | | | | Q6 | | | | Q7 | | | | Q8 | | | | Q9 | | | | Q10 | | | | Q11 | | | | Overall quality |
| --- | --- | --- | --- | --- | --- | --- | --- | --- | --- | --- | --- | --- | --- | --- | --- | --- | --- | --- | --- | --- | --- | --- | --- | --- | --- | --- | --- | --- | --- | --- | --- | --- | --- | --- | --- | --- | --- | --- | --- | --- | --- | --- | --- | --- | --- |
|  | Y | N | U | NA | Y | N | U | NA | Y | N | U | NA | Y | N | U | NA | Y | N | U | NA | Y | N | U | NA | Y | N | U | NA | Y | N | U | NA | Y | N | U | NA | Y | N | U | NA | Y | N | U | NA |  |
| Wondie et. al, 2023. |  |  |  | **√** |  |  |  | **√** | **√** |  |  |  | **√** |  |  |  | **√** |  |  |  | **√** |  |  |  | **√** |  |  |  | **√** |  |  |  | **√** |  |  |  | **√** |  |  |  | **√** |  |  |  | 9/11(81.81%) |
| Woelile, et,, 2021. |  |  |  | **√** |  |  |  | **√** | **√** |  |  |  | **√** |  |  |  | **√** |  |  |  | **√** |  |  |  | **√** |  |  |  | **√** |  |  |  | **√** |  |  |  | **√** |  |  |  | **√** |  |  |  | 9/11(81.81%) |
| Kebede, et, a1 2022 |  |  |  | **√** |  |  |  | **√** | **√** |  |  |  | **√** |  |  |  | **√** |  |  |  | **√** |  |  |  | **√** |  |  |  | **√** |  |  |  | **√** |  |  |  | **√** |  |  |  | **√** |  |  |  | 9/11(81.81%) |
| Negussie, et, al 2024 |  |  |  | **√** |  |  |  | **√** | **√** |  |  |  | **√** |  |  |  | **√** |  |  |  | **√** |  |  |  | **√** |  |  |  | **√** |  |  |  | **√** |  |  |  | **√** |  |  |  | **√** |  |  |  | 9/11(81.81%) |
| Tessema, et, al 2022 |  |  |  | **√** |  |  |  | **√** | **√** |  |  |  | **√** |  |  |  | **√** |  |  |  | **√** |  |  |  | **√** |  |  |  | **√** |  |  |  | **√** |  |  |  | **√** |  |  |  | **√** |  |  |  | 9/11(81.81%) |
| Alemaw, et al. 2022 |  |  |  |  | **√** |  |  |  | **√** |  |  |  | **√** |  |  |  | **√** |  |  |  | **√** |  |  |  | **√** |  |  |  | **√** |  |  |  | **√** |  |  |  | **√** |  |  |  | **√** |  |  |  | 9/11(81.81%) |
| Debere, et, al 2022 |  |  |  | **√** |  |  |  | **√** | **√** |  |  |  | **√** |  |  |  | **√** |  |  |  | **√** |  |  |  | **√** |  |  |  | **√** |  |  |  | **√** |  |  |  | **√** |  |  |  | **√** |  |  |  | 9/11(81.81%) |
| Dessu, et. Al 2020 |  |  |  | **√** |  |  |  | **√** | **√** |  |  |  | **√** |  |  |  | **√** |  |  |  | **√** |  |  |  | **√** |  |  |  | **√** |  |  |  | **√** |  |  |  | **√** |  |  |  | **√** |  |  |  | 9/11(81.81%) |
| Birhanu, et, al 2023 |  |  |  | **√** |  |  |  | **√** | **√** |  |  |  | **√** |  |  |  | **√** |  |  |  | **√** |  |  |  | **√** |  |  |  | **√** |  |  |  | **√** |  |  |  | **√** |  |  |  | **√** |  |  |  | 9/11(81.81%) |
| Genie, et, al 2022 |  |  |  | **√** |  |  |  | **√** | **√** |  |  |  | **√** |  |  |  | **√** |  |  |  | **√** |  |  |  |  |  |  |  | **√** |  |  |  | **√** |  |  |  | **√** |  |  |  | **√** |  |  |  | 9/11(81.81%) |
| Mislu , et, al  2024 |  |  |  | **√** |  |  |  | **√** | **√** |  |  |  | **√** |  |  |  | **√** |  |  |  | **√** |  |  |  | **√** |  |  |  | **√** |  |  |  | **√** |  |  |  | **√** |  |  |  | **√** |  |  |  | 9/11(81.81%) |

S3 Table B: Quality assessment for included studies in meta-analysis for cross-sectional design

| Author, Year | Q1 | | | Q2 | | | | Q3 | | | | Q4 | | | | Q5 | | | | Q6 | | | | Q7 | | | | Q8 | | | | Overall quality in % |
| --- | --- | --- | --- | --- | --- | --- | --- | --- | --- | --- | --- | --- | --- | --- | --- | --- | --- | --- | --- | --- | --- | --- | --- | --- | --- | --- | --- | --- | --- | --- | --- | --- |
|  | N | U | NA | Y | N | U | NA | Y | N | U | NA | Y | N | U | NA | Y | N | U | NA | Y | N | U | NA | Y | N | U | NA | Y | N | U | NA |  |
| Eshete, et, al, 2019. | **√** |  |  | **√** |  |  |  | **√** |  |  |  | **√** |  |  |  | **√** |  |  |  | **√** |  |  |  | **√** |  |  |  | **√** |  |  |  | 8/8 (100) |
| Abraham 2021 | **√** |  |  | **√** |  |  |  | **√** |  |  |  | **√** |  |  |  | **√** |  |  |  | **√** |  |  |  | **√** |  |  |  | **√** |  |  |  | 8/8 (100) |
| Gedamu, et al. 2019 | **√** |  |  | **√** |  |  |  | **√** |  |  |  |  |  | √ |  | **√** |  |  |  | **√** |  |  |  | **√** |  |  |  | **√** |  |  |  | 7/8 (87.5) |
| Worku 1999 | **√** |  |  | **√** |  |  |  | **√** |  |  |  |  |  | **√** |  | **√** |  |  |  | **√** |  |  |  | **√** |  |  |  | **√** |  |  |  | 7/8 (87.5%) |

*Y=yes, N=no, U=unclear, NA=not applicable, <60%=low, 60-80%=medium, >80%=high quality.
