# Supplementary material for: Incidence of mortality and its predictors among low birth weight neonates in Ethiopia: Systematic review and meta-analysis
Source: PLoS One. 2026 Jul 29;21(7):e0344213. doi: 10.1371/journal.pone.0344213 (PMC13419215; doi:10.1371/journal.pone.0344213)
Supplement: S4 Table — (DOCX) [file pone.0344213.s004.docx]

**S4: Table Certainty of evidence according to Grading of Recommendations Assessment, Development, and Evaluation (GRADE) Approach.**

| **Outcome/**  **/Predictor** | **Studies (n)** | **Effect (95% CI)** | **Certainty** | **Rationale (5 GRADE criteria)** |
| --- | --- | --- | --- | --- |
| **Mortality Proportion** | 15 | 26.67% (21.83-31.51%) | ⊕⊕⊝⊝ **LOW** | RoB: none; **Inconsistency: serious** (I²=96%); Indirectness: none; **Imprecision: some** (wide CI); **Pub. bias: serious** (Egger's p=0.026). |
| **Incidence Rate** | 10 | 36.31/1000d (26.37-46.25) | ⊕⊕⊝⊝ **LOW** | RoB: none; **Inconsistency: serious** (I²=97%); Indirectness: none; **Imprecision: serious** (wide CI); **Pub. bias: serious** (p=0.005). |
| **Preeclampsia** | 6 | AHR 1.38 (1.09-1.75) | ⊕⊕⊕⊝ **MOD** | RoB: none; Inconsistency: none (I²=38.8%); Indirectness: none; Imprecision: none; Pub. bias: none. |
| **RDS** | 7 | AHR 1.67 (1.25-2.23) | ⊕⊕⊝⊝ **LOW** | RoB: none; **Inconsistency: serious** (I²=64.8%); Indirectness: none; Imprecision: none; Pub. bias: none. ​ |
| **PNA** | 6 | AHR 1.66 (1.35-2.05) | ⊕⊕⊕⊝ **MOD** | RoB: none; Inconsistency: none (I²=0%); Indirectness: none; Imprecision: none; Pub. bias: none. |
| **Sepsis** | 4 | AHR 2.04 (1.59-2.63) | ⊕⊕⊝⊝ **LOW** | RoB: none; Inconsistency: some (I²=48.9%); Indirectness: none; Imprecision: some; Pub. bias: none. |
| **Non-breastfeeding** | 4 | AHR 5.16 (2.61-9.96) | ⊕⊕⊝⊝ **LOW** | RoB: none; **Inconsistency: serious** (I²=66.2%); Indirectness: none; **Imprecision: serious** (wide CI); Pub. bias: none.​ |
| **Not using KMC** | 4 | AHR 4.46 (2.19-9.10) | ⊕⊕⊝⊝ **LOW** | RoB: none; Inconsistency: some (I²=46.1%); Indirectness: none; **Imprecision: serious** (wide CI); Pub. bias: none. ​ |
| **Hypothermia** | 5 | AHR 1.24 (1.01-1.53) | ⊕⊕⊕⊝ **MOD** | RoB: none; Inconsistency: none (I²=0%); Indirectness: none; Imprecision: none; Pub. bias: none. ​ |
| **Prematurity** | 6 | AHR 1.55 (1.07-2.24) | ⊕⊕⊝⊝ **LOW** | RoB: none; Inconsistency: some (I²=53.5%); Indirectness: none; **Imprecision: some** (CI ↙1); Pub. bias: none. |
| **ELBW** | 8 | AHR 3.67 (2.55-5.29) | ⊕⊕⊕⊝ **MOD** | RoB: none; Inconsistency: some (I²=49.8%); Indirectness: none; Imprecision: none; Pub. bias: none. |
| **VLBW** | 9 | AHR 1.75 (1.44-2.12) | ⊕⊕⊕⊝ **MOD** | RoB: none; Inconsistency: none (I²=32.8%); Indirectness: none; Imprecision: none; Pub. bias: none. |

**Abbreviations: RoB: Risk of Bias, Pub.Bias: Publication bias.**
