# Supplementary material for: Incidence of mortality and its predictors among low birth weight neonates in Ethiopia: Systematic review and meta-analysis
Source: PLoS One. 2026 Jul 29;21(7):e0344213. doi: 10.1371/journal.pone.0344213 (PMC13419215; doi:10.1371/journal.pone.0344213)
Supplement: Attachment — Submitted filename: Response to Reviewers.docx [file pone.0344213.s006.docx]

**Authors' response to the Editor, and Reviewers' concern.**

Title: **Incidence of Mortality and its Predictors among Low Birth Weight Neonates in Ethiopia: Systematic Review and Meta-Analysis**.

**Manuscript ID: PONE-D-24-28796.**

To: PLOS ONE JOURNAL

Date: January 24, 2026.

**Subject: Revision of the manuscript.**

Thank you very much, dear editor and reviewers, for your valuable and constructive suggestions and comments. We found that the comments are very helpful and constructive to further improve the manuscript. This is our point-by-point response to the editor and reviewers’ suggestions and concerns about the manuscript entitled “Incidence of Mortality and its Predictors among Low Birth Weight Neonates in Ethiopia: Systematic Review and Meta-Analysis,” which has a manuscript ID of “PONE-D-24-28796” given by the journal. It is known that the manuscript has been reviewed by reviewers and sent back to the authors for revision and resubmission. As authors of this manuscript, the comments and concerns raised by the reviewers and editor are highly important, enabling us to improve the quality and plausibility of the manuscript. To do so, we have addressed all of the reviewers' concerns point by point. Therefore, we are very pleased to resubmit the revised version of the manuscript for further revision process and facilitation of its publication on PLOS ONE.

We look forward to hearing from you at your earliest convenience.

With best regards.

Corresponding Author

Wubet Tazeb Wondie	E-mail: wubettazeb27@gmail.com.

On behalf of the co-authors.

**Point-by-Point Response Letter**

Dear editorial office of PLOS ONE, we have presented our point-by-point response in a way that the reviewers' concern is depicted first, and the authors' response has been given immediately next to it.

**Reviewer 1: Concerns and Authors’ Response.**

Reviewer #1: Dear Author,

Your manuscript looks good, and you did an extensive literature search for this review article.

**Author's response:** Dear respected reviewer, we thank you for your encouraging words.

My comments

1. **Concern 1:** 1. Reference no. 44,48,49,51,75 should be re-write in Vancouver style referencing.

**Author's response:** Dear reviewer, we thank you for your concern. We have accepted and corrected.

Dear respected reviewer, We are very grateful for your immense experience and thorough review of the manuscript. We found all of your comments constructive and valuable for improving this manuscript for publication. If something is unclear/wrong, please let me know again!

**Reviewer 2: Concerns and Authors’ Response.**

**Concern 1:** 1. Background: Respiratory Distress Syndrome (RDS) (23), mode of delivery (23): What is the relevance of citing reference 23 two times?

**Author's response:** Dear reviewer, we thank you for your valuable suggestions. We have accepted and corrected.

2. **Methods:**

**Concern 2:** You have to clearly state the searching date (when was your last searching date?)

**Author's response:** Dear esteemed reviewer, thank you for your concern. We have accepted and corrected.

**Concern 3:** How did you assess the certainty of evidence?

**Author's response:** Dear respected reviewer, we thank you for your valuable concern. We assessed the methodological quality of included studies using the JBI checklist, with all studies rated high quality (see Supplementary Table S3). Additionally, the certainty of evidence was assessed using the **GRADE approach.** We updated the methods and results section (see method section on page 7 and result section on page 18, “supplementary file 4”).

**3. Result section**

**Concern 4:** Why you only include 10 studies to calculate the pooled incidence rate, and other systematic review in Africa (Incidence and predictors of mortality low birth weight neonates in Africa) included 14 studies from Ethiopia?

**Author's response:** Dear respected reviewer, thank you for your valuable concern. In our study, we included a total of 15 primary studies to report the proportion of death and 10 primary studies to estimate the incidence rate of death among low birth weight (LBW) neonates. However, the study conducted in Africa by Mengstie et al. (2025). <https://www.frontiersin.org/journals/pediatrics/articles/10.3389/fped.2025.1458871/full#B14> has several reporting issues in its incidence rate of mortality. Key problems include:

1. Incorporating studies that report the prevalence of LBW as mortality (e.g., Zeleke et al., available at “<https://pubmed.ncbi.nlm.nih.gov/22826729/>”.
2. Treating studies reporting the proportion of death as incidence rates (e.g., Genie et al. <https://pubmed.ncbi.nlm.nih.gov/35803633/> and Eshete et al. <https://pubmed.ncbi.nlm.nih.gov/31223314/>).
3. In addition to the aforementioned problems. They included studies that assess the mortality of low birth weight neonates beyond the neonatal period, Gebeerekidan AB. <https://www.researchsquare.com/article/rs-4219427/v1>.

Our study focuses specifically on LBW neonates, as reflected in the title: "Incidence and proportion of mortality and predictors among low birth weight neonates." Thus, we restricted inclusion to studies reporting incidence rate and proportion of mortality up to the neonatal period only. Pooling LBW prevalence, death proportions as incidence rates, and post-neonatal mortality data inevitably leads to biased estimates.

For these reasons, we selected 10 studies reporting incidence rates, meaning studies like Zeleke et al., Genie et al., and Eshete et al. were not included. On the other hand, 15 for reporting pooled proportions of death, we incorporated 15 studies, which did include Zeleke et al., Genie et al., and Eshete et al., were included. I wonder if you could review these primary studies.

Dear reviewer, we thank you for your valuable concerns and time. All of your concerns were constructive, and they helped us to improve our manuscript for publication. If something is unclear/wrong, please let me know again!

We author would like to thank the Editor and the reviewer for their constructive concerns.
