# Supplementary material for: Incidence of mortality and its predictors among low birth weight neonates in Ethiopia: Systematic review and meta-analysis
Source: PLoS One. 2026 Jul 29;21(7):e0344213. doi: 10.1371/journal.pone.0344213 (PMC13419215; doi:10.1371/journal.pone.0344213)
Supplement: Peer review history

# Peer review: Decision Letter 0

**DOI:** 10.1371/journal.pone.0344213.r001

**Authors:** Sanjoy Dey

19 Nov 2025

PONE-D-24-28796

Incidence and proportion of mortality and its predictor among low birth weight neonates in Ethiopia: A systematic review and meta-analysis.

PLOS ONE

Dear Dr. Wubet Tazeb Wondie

Thank you for submitting your manuscript to PLOS ONE. After careful consideration, we feel that it has merit but does not fully meet PLOS ONE’s publication criteria as it currently stands. Therefore, we invite you to submit a revised version of the manuscript that addresses the points raised during the review process.

Please submit your revised manuscript by Jan 03 2026 11:59PM. If you will need more time than this to complete your revisions, please reply to this message or contact the journal office at plosone@plos.org . When you're ready to submit your revision, log on to https://www.editorialmanager.com/pone/ and select the 'Submissions Needing Revision' folder to locate your manuscript file.

Please include the following items when submitting your revised manuscript:

- A rebuttal letter that responds to each point raised by the academic editor and reviewer(s). You should upload this letter as a separate file labeled 'Response to Reviewers'.
- A marked-up copy of your manuscript that highlights changes made to the original version. You should upload this as a separate file labeled 'Revised Manuscript with Track Changes'.
- An unmarked version of your revised paper without tracked changes. You should upload this as a separate file labeled 'Manuscript'.

-->If you would like to make changes to your financial disclosure, please include your updated statement in your cover letter. Guidelines for resubmitting your figure files are available below the reviewer comments at the end of this letter.

We look forward to receiving your revised manuscript.

Kind regards,

Sanjoy Kumer Dey, M.D

Academic Editor

PLOS ONE

Journal Requirements:

When submitting your revision, we need you to address these additional requirements.

1. Please ensure that your manuscript meets PLOS ONE's style requirements, including those for file naming. The PLOS ONE style templates can be found at

https://journals.plos.org/plosone/s/file?id=wjVg/PLOSOne_formatting_sample_main_body.pdf and

https://journals.plos.org/plosone/s/file?id=ba62/PLOSOne_formatting_sample_title_authors_affiliations.pdf .

2. If the reviewer comments include a recommendation to cite specific previously published works, please review and evaluate these publications to determine whether they are relevant and should be cited. There is no requirement to cite these works unless the editor has indicated otherwise.

3. Please review your reference list to ensure that it is complete and correct. If you have cited papers that have been retracted, please include the rationale for doing so in the manuscript text, or remove these references and replace them with relevant current references. Any changes to the reference list should be mentioned in the rebuttal letter that accompanies your revised manuscript. If you need to cite a retracted article, indicate the article’s retracted status in the References list and also include a citation and full reference for the retraction notice.

[Note: HTML markup is below. Please do not edit.]

Reviewers' comments:

Reviewer's Responses to Questions

**Comments to the Author**

1. Is the manuscript technically sound, and do the data support the conclusions?

The manuscript must describe a technically sound piece of scientific research with data that supports the conclusions. Experiments must have been conducted rigorously, with appropriate controls, replication, and sample sizes. The conclusions must be drawn appropriately based on the data presented.

Reviewer #1: Yes

Reviewer #2: Partly

**********

2. Has the statistical analysis been performed appropriately and rigorously?

Reviewer #1: Yes

Reviewer #2: Yes

**********

3. Have the authors made all data underlying the findings in their manuscript fully available?

The PLOS Data policy requires authors to make all data underlying the findings described in their manuscript fully available without restriction, with rare exception (please refer to the Data Availability Statement in the manuscript PDF file). The data should be provided as part of the manuscript or its supporting information, or deposited to a public repository. For example, in addition to summary statistics, the data points behind means, medians and variance measures should be available. If there are restrictions on publicly sharing data—e.g. participant privacy or use of data from a third party—those must be specified.

Reviewer #1: Yes

Reviewer #2: Yes

**********

4. Is the manuscript presented in an intelligible fashion and written in standard English?

PLOS ONE does not copyedit accepted manuscripts, so the language in submitted articles must be clear, correct, and unambiguous. Any typographical or grammatical errors should be corrected at revision, so please note any specific errors here.

Reviewer #1: Yes

Reviewer #2: Yes

**********

5. Review Comments to the Author

Please use the space provided to explain your answers to the questions above. You may also include additional comments for the author, including concerns about dual publication, research ethics, or publication ethics. (Please upload your review as an attachment if it exceeds 20,000 characters)

Reviewer #1: Dear Author,

Your mansucript looks good and you did extensive literature search for this reveiw article. My comments

1. Reference no. 44,48,49,51,75 should be re-write in Vancouver style referencing.

Reviewer #2: 1.Back ground: Respiratory Distress Syndrome (RDS) (23), mode of delivery (23): what is the relevance of citing reference 23 two times?

2.Methods:

•You have to clearly state the searching date (when was your last searching date?)

•How did you assess the certainty of evidence?

3.Result section

•Why you only included 10 studies to calculate the pooled incidence rate, and other systematic review in Africa (Incidence and predictors of mortality low birth weight neonates in Africa) included 14 studies from Ethiopia?

**********

6. PLOS authors have the option to publish the peer review history of their article (what does this mean? ). If published, this will include your full peer review and any attached files.

If you choose “no”, your identity will remain anonymous but your review may still be made public.

**Do you want your identity to be public for this peer review?**  For information about this choice, including consent withdrawal, please see our Privacy Policy .

Reviewer #1: **Yes:**   Prof. Dr Sunil Raja Manandhar

Reviewer #2: No

**********

[NOTE: If reviewer comments were submitted as an attachment file, they will be attached to this email and accessible via the submission site. Please log into your account, locate the manuscript record, and check for the action link "View Attachments". If this link does not appear, there are no attachment files.]

To ensure your figures meet our technical requirements, please review our figure guidelines: https://journals.plos.org/plosone/s/figures

You may also use PLOS’s free figure tool, NAAS, to help you prepare publication quality figures: https://journals.plos.org/plosone/s/figures#loc-tools-for-figure-preparation.

NAAS will assess whether your figures meet our technical requirements by comparing each figure against our figure specifications.

# Author comment: Author response to Decision Letter 1

**DOI:** 10.1371/journal.pone.0344213.r002

31 Jan 2026

Authors' response to the Editor, and Reviewers' concern.

Title: Incidence of Mortality and its Predictors among Low Birth Weight Neonates in Ethiopia: Systematic Review and Meta-Analysis.

Manuscript ID: PONE-D-24-28796.

To: PLOS ONE JOURNAL

Date: January 24, 2026.

Subject: Revision of the manuscript.

Thank you very much, dear editor and reviewers, for your valuable and constructive suggestions and comments. We found that the comments are very helpful and constructive to further improve the manuscript. This is our point-by-point response to the editor and reviewers’ suggestions and concerns about the manuscript entitled “Incidence of Mortality and its Predictors among Low Birth Weight Neonates in Ethiopia: Systematic Review and Meta-Analysis,” which has a manuscript ID of “PONE-D-24-28796” given by the journal. It is known that the manuscript has been reviewed by reviewers and sent back to the authors for revision and resubmission. As authors of this manuscript, the comments and concerns raised by the reviewers and editor are highly important, enabling us to improve the quality and plausibility of the manuscript. To do so, we have addressed all of the reviewers' concerns point by point. Therefore, we are very pleased to resubmit the revised version of the manuscript for further revision process and facilitation of its publication on PLOS ONE.

We look forward to hearing from you at your earliest convenience.

With best regards.

Corresponding Author

Wubet Tazeb Wondie E-mail: wubettazeb27@gmail.com.

On behalf of the co-authors.

Point-by-Point Response Letter

Dear editorial office of PLOS ONE, we have presented our point-by-point response in a way that the reviewers' concern is depicted first, and the authors' response has been given immediately next to it.

Reviewer 1: Concerns and Authors’ Response.

Reviewer #1: Dear Author,

Your manuscript looks good, and you did an extensive literature search for this review article.

Author's response: Dear respected reviewer, we thank you for your encouraging words.

My comments

1. Concern 1: 1. Reference no. 44,48,49,51,75 should be re-write in Vancouver style referencing.

Author's response: Dear reviewer, we thank you for your concern. We have accepted and corrected.

Dear respected reviewer, We are very grateful for your immense experience and thorough review of the manuscript. We found all of your comments constructive and valuable for improving this manuscript for publication. If something is unclear/wrong, please let me know again!

Reviewer 2: Concerns and Authors’ Response.

Concern 1: 1. Background: Respiratory Distress Syndrome (RDS) (23), mode of delivery (23): What is the relevance of citing reference 23 two times?

Author's response: Dear reviewer, we thank you for your valuable suggestions. We have accepted and corrected.

2. Methods:

Concern 2: You have to clearly state the searching date (when was your last searching date?)

Author's response: Dear esteemed reviewer, thank you for your concern. We have accepted and corrected.

Concern 3: How did you assess the certainty of evidence?

Author's response: Dear respected reviewer, we thank you for your valuable concern. We assessed the methodological quality of included studies using the JBI checklist, with all studies rated high quality (see Supplementary Table S3). Additionally, the certainty of evidence was assessed using the GRADE approach. We updated the methods and results section (see method section on page 7 and result section on page 18, “supplementary file 4”).

3. Result section

Concern 4: Why you only include 10 studies to calculate the pooled incidence rate, and other systematic review in Africa (Incidence and predictors of mortality low birth weight neonates in Africa) included 14 studies from Ethiopia?

Author's response: Dear respected reviewer, thank you for your valuable concern. In our study, we included a total of 15 primary studies to report the proportion of death and 10 primary studies to estimate the incidence rate of death among low birth weight (LBW) neonates. However, the study conducted in Africa by Mengstie et al. (2025). https://www.frontiersin.org/journals/pediatrics/articles/10.3389/fped.2025.1458871/full#B14 has several reporting issues in its incidence rate of mortality. Key problems include:

1. Incorporating studies that report the prevalence of LBW as mortality (e.g., Zeleke et al., available at “https://pubmed.ncbi.nlm.nih.gov/22826729/”.

2. Treating studies reporting the proportion of death as incidence rates (e.g., Genie et al. https://pubmed.ncbi.nlm.nih.gov/35803633/ and Eshete et al. https://pubmed.ncbi.nlm.nih.gov/31223314/).

3. In addition to the aforementioned problems. They included studies that assess the mortality of low birth weight neonates beyond the neonatal period, Gebeerekidan AB. https://www.researchsquare.com/article/rs-4219427/v1.

Our study focuses specifically on LBW neonates, as reflected in the title: "Incidence and proportion of mortality and predictors among low birth weight neonates." Thus, we restricted inclusion to studies reporting incidence rate and proportion of mortality up to the neonatal period only. Pooling LBW prevalence, death proportions as incidence rates, and post-neonatal mortality data inevitably leads to biased estimates.

For these reasons, we selected 10 studies reporting incidence rates, meaning studies like Zeleke et al., Genie et al., and Eshete et al. were not included. On the other hand, 15 for reporting pooled proportions of death, we incorporated 15 studies, which did include Zeleke et al., Genie et al., and Eshete et al., were included. I wonder if you could review these primary studies.

Dear reviewer, we thank you for your valuable concerns and time. All of your concerns were constructive, and they helped us to improve our manuscript for publication. If something is unclear/wrong, please let me know again!

We author would like to thank the Editor and the reviewer for their constructive concerns.

Attachment Submitted filename: Response to Reviewers.docx

# Peer review: Decision Letter 1

**DOI:** 10.1371/journal.pone.0344213.r003

**Authors:** Sanjoy Dey

17 Feb 2026

Incidence of mortality and its predictor among low birth weight neonates in Ethiopia: A systematic review and meta-analysis.

PONE-D-24-28796R1

Dear Dr. Wubet Tazeb Wondie

We’re pleased to inform you that your manuscript has been judged scientifically suitable for publication and will be formally accepted for publication once it meets all outstanding technical requirements.

Within one week, you’ll receive an e-mail detailing the required amendments. When these have been addressed, you’ll receive a formal acceptance letter and your manuscript will be scheduled for publication.

An invoice will be generated when your article is formally accepted. Please note, if your institution has a publishing partnership with PLOS and your article meets the relevant criteria, all or part of your publication costs will be covered. Please make sure your user information is up-to-date by logging into Editorial Manager at Editorial Manager®  and clicking the ‘Update My Information' link at the top of the page. For questions related to billing, please contact billing support .

If your institution or institutions have a press office, please notify them about your upcoming paper to help maximize its impact. If they’ll be preparing press materials, please inform our press team as soon as possible -- no later than 48 hours after receiving the formal acceptance. Your manuscript will remain under strict press embargo until 2 pm Eastern Time on the date of publication. For more information, please contact onepress@plos.org.

Kind regards,

Sanjoy Kumer Dey, M.D

Academic Editor

PLOS One

Additional Editor Comments (optional):

Reviewers' comments:

Reviewer's Responses to Questions

**Comments to the Author**

1. If the authors have adequately addressed your comments raised in a previous round of review and you feel that this manuscript is now acceptable for publication, you may indicate that here to bypass the “Comments to the Author” section, enter your conflict of interest statement in the “Confidential to Editor” section, and submit your "Accept" recommendation.

Reviewer #2: All comments have been addressed

**********

2. Is the manuscript technically sound, and do the data support the conclusions?

The manuscript must describe a technically sound piece of scientific research with data that supports the conclusions. Experiments must have been conducted rigorously, with appropriate controls, replication, and sample sizes. The conclusions must be drawn appropriately based on the data presented.

Reviewer #2: Partly

**********

3. Has the statistical analysis been performed appropriately and rigorously?

Reviewer #2: Yes

**********

4. Have the authors made all data underlying the findings in their manuscript fully available?

The PLOS Data policy requires authors to make all data underlying the findings described in their manuscript fully available without restriction, with rare exception (please refer to the Data Availability Statement in the manuscript PDF file). The data should be provided as part of the manuscript or its supporting information, or deposited to a public repository. For example, in addition to summary statistics, the data points behind means, medians and variance measures should be available. If there are restrictions on publicly sharing data—e.g. participant privacy or use of data from a third party—those must be specified.

Reviewer #2: Yes

**********

5. Is the manuscript presented in an intelligible fashion and written in standard English?

PLOS ONE does not copyedit accepted manuscripts, so the language in submitted articles must be clear, correct, and unambiguous. Any typographical or grammatical errors should be corrected at revision, so please note any specific errors here.

Reviewer #2: Yes

**********

6. Review Comments to the Author

Please use the space provided to explain your answers to the questions above. You may also include additional comments for the author, including concerns about dual publication, research ethics, or publication ethics. (Please upload your review as an attachment if it exceeds 20,000 characters)

Reviewer #2: (No Response)

**********

7. PLOS authors have the option to publish the peer review history of their article (what does this mean? ). If published, this will include your full peer review and any attached files.

If you choose “no”, your identity will remain anonymous but your review may still be made public.

**Do you want your identity to be public for this peer review?**  For information about this choice, including consent withdrawal, please see our Privacy Policy .

Reviewer #2: No

**********

# Editor report: Acceptance letter

**DOI:** 10.1371/journal.pone.0344213.r004

**Authors:** Sanjoy Dey

PONE-D-24-28796R1

PLOS One

Dear Dr. Wondie,

I'm pleased to inform you that your manuscript has been deemed suitable for publication in PLOS One. Congratulations! Your manuscript is now being handed over to our production team.

At this stage, our production department will prepare your paper for publication. This includes ensuring the following:

* All references, tables, and figures are properly cited

* All relevant supporting information is included in the manuscript submission,

* There are no issues that prevent the paper from being properly typeset

You will receive further instructions from the production team, including instructions on how to review your proof when it is ready. Please keep in mind that we are working through a large volume of accepted articles, so please give us a few days to review your paper and let you know the next and final steps.

Lastly, if your institution or institutions have a press office, please let them know about your upcoming paper now to help maximize its impact. If they'll be preparing press materials, please inform our press team within the next 48 hours. Your manuscript will remain under strict press embargo until 2 pm Eastern Time on the date of publication. For more information, please contact onepress@plos.org.

You will receive an invoice from PLOS for your publication fee after your manuscript has reached the completed accept phase. If you receive an email requesting payment before acceptance or for any other service, this may be a phishing scheme. Learn how to identify phishing emails and protect your accounts at https://explore.plos.org/phishing.

If we can help with anything else, please email us at customercare@plos.org.

Thank you for submitting your work to PLOS ONE and supporting open access.

Kind regards,

PLOS ONE Editorial Office Staff

on behalf of

Dr. Sanjoy Kumer Dey

Academic Editor

PLOS One
